# A New Monstrosity

**Published:** 1840-07

**Authors:** 


*Notice of a New Monstrosity; A portion of a Fœtus living upon the Testicle.* By M. Velpeau.—The case on which I propose to engage the Academy to-day, is one of the most strange that the sciences of observation have yet had to consider; interesting at once to surgery, pathology, anatomy, generation, physiology in general, it appears to be without parallel among known facts. It relates to a living portion of a fœtus fixed in the testicle of an adult, where it seems to have been developed and to have lived since his birth. This is a peculiarity so contrary to what we know, and is at first glance so incomprehensible, that one might be justified in doubting its existence if I did not possess the substantial proof of it in the preparations here presented, and if the patient and the tumour had not been observed by several hundreds of practitioners and students, and if the operation had not been performed in the presence of 500 persons. The case is, in a few words, as follows:

A young man, named Gallochat, of Esterney, aged 27, of a good constitution, and who had never suffered from any severe disease, was sent, in the middle of January, to M. Andral, who at once passed him over to my division in the Hopital de la Charite.

On examination, I found that the patient had a tumour, nearly as large as a fist, on the right side of the scrotum. It appeared unconnected with the substance of the testicle; the skin over it presented no analogy to that of the scrotum, and it did not appear to me to belong to any known class of tumours. Although several surgeons thought it might be referred, some to the cancerous tumours, some to the fibrous, and some to the tuberculous class, I did not think it possible to adopt their opinion. Observing, moreover, that its origin dated back to the patient's birth, that it was not perceived at its commencement, that it had never produced any pain, that no pathological process had been set up in it, and that it could be cut, or pricked, or pierced through and through, without causing the least suffering; taking notice also of the aspect of the skin which covered its external surface, of its elasticity, of the indurations which it presented internally, of a tuft of hair which came from a kind of ulcer at its posterior part, of a reddish tubercle at the bottom of another opening anteriorly, and of a glairy or grumous matter which the patient had sometimes discharged; I came to the idea that it was a *fœtal tumor*, *a product of conception.*

Wishing to obtain exact information on the earliest history of so singular a production, I wrote to M. Senoble, physician at Esternay, who answered me thus: "At the age of about four months, the mother of Gallochat came to show me her child; he then had a tumour, or merely a swelling of the scrotum, which I found to be only a pneumatocele. Some months afterwards, I found, on examining him again, a small inflamed tumour, which appeared to me to be a slight phlegmon, and which yielded to simple emollient local applications. I heard no more of him till at the end of three or four years, when I learned that the child's tumour still continued enlarging." Now although these details were very incomplete, they yet strengthened me in my first opinion; which seemed so singular to those to whom I mentioned it, that I alone held it. I therefore planned the removal of the tumour without taking away the testicle, intending to perform a kind of *Cæsarean operation on the man.* The details of the proceeding belong entirely to surgery, and need not now occupy me; it may be sufficient to state that its results were satisfactory.

The examination of the tumour has enabled me to detect nearly all the anatomical elements of the body of a mammal. Thus, its external layer is evidently cutaneous; the greater part of its substance is a mixture of lamellæ and fibres which give the idea of the cellular, adipose, muscular, and fibrous tissues. In its interior, we found two small cysts filled with matter like albumen or the vitreous humour of the eye; another cyst, as large as a partridge's egg, contained a greenish yellow and semi-liquid matter like meconium; in a fourth sac there was a grumous substance, of a dirty-yellow colour, concrete, and surrounded with hair. The substance from this last sac, when analyzed and examined with the microscope, presented all the characters of sebaceous matter and scales of epidermis. The hairs did not appear to have any bulbs at their bases. The tuft of hair which was seen externally, protruded from one of these cysts—from that which was filled with greenish matter; and the opening in it had some analogy with an anus.

Lastly, in the midst of all these elements, we found numerous portions of the skeleton perfectly organized, evidently belonging (as any one may convince himself by examining the preparation) to true bones, and not to accidental productions. These bones, which were every where enveloped by a sort of periosteum, and of which the several pieces were moveable upon each other, and had distinct articulations, may be divided into three sets. The first group is essentially composed of three pieces, in which I thought I could recognize the clavicle, the scapula, and a part of the humerus. The second group, much larger than the preceding, appears to belong to the pelvis, or perhaps to the base of the skull; the body of the sphenoid, or else the sacrum, forms the central portion. Lastly, the third series seems to comprehend portions of vertebræ and fragments of undetermined bones.

Whatever be the name that the different portions I have pointed out may deserve, certain it is that they belong to a product of conception, and to a fœtus already far advanced in its development. They are before the Academy, and the correctness of the fact is absolutely incontrovertible. In the monstrosity by inclusion, as it is called, which has been described by Dupuytren, Geoffroy, and Olivier, one of the fœtuses absorbed by the other has always appeared surrounded by a cyst, and in the condition of a foreign body in the tissues of the fœtus which has continued alive. In the cases related by Saint-Donat, Prochaska, and others, of the debris of fœtuses contained in the scrotum, there have always been encysted tumours, necrosed bones, and organized parts destroyed by suppuration and in a state of decomposition. In this subject, on the contrary, every thing has continued to live. The abnormal tumor had its own proper colour, consistence, and sensibility, entirely independent of the individual who supported it; a clear well-defined line separated the integuments of its skin from the scrotum. I pinched it with all possible force; I pricked it with various instruments; the young man himself several times ran a knife into it, without feeling the least painful sensations; and yet all the wounds that were made in it bled abundantly, inflamed, and cicatrized, like those of any other part of the body, and nothing indicated in it the least diseased condition. The substances, and all the elements that were found in it, gave the idea of normal tissues or products, and we were quite unable to discover the existence of the least drop of pus, or of any carious or necrosed bone, any altered cartilage, or the least fungous production.

When, on the other hand, one observes that the tumor was as large as a fist—that the surgeon who saw the child when four months old scarcely took notice of it, and that he took it at first for a pneumatocele, and then for a little phlegmon, which terminated by resolution—it is difficult to believe that its volume was as considerable at the birth of the patient as it was the time when I first saw it. Such a mass in an infant would certainly have attracted great attention both from the physician and the family. We must remember, moreover, that, according to M. Senoble's statement, the tumor continued to grow at least up to the age of six or seven years, and that the young man, who says that it has always had the same appearance, can scarcely charge his memory so far back as that time of his life: we must therefore conclude that the portions of the fœtus which I have described have lived and been developed simultaneously with the individual who bore them, and that there were thus two beings united to one another.

Now how could this take place? Did a part of the fœtus, the remainder of which has disappeared, become attached, during intrauterine life, to the scrotum, in such a manner as to remain there in the form of a graft?—or can this be the remains of a fætus which at first passed into the abdomen of another, and then descended by the tunica vaginalis, and has at last worn away from within outwards the envelopes of the scrotum?—or, lastly, have we here a creation, the unaided product, of the testicle? But I desist; these are delicate questions in high physiology and in transcendental anatomy, which I am neither able nor willing to broach till the preparations which suggest them have been submitted to the judgment of the Academy.—*London, from Paris Medical Gazette, Feb.* 15, 1840.—*Medical Examiner, June.*

